# A CRISPR knockout screen reveals new regulators of canonical Wnt signaling

**DOI:** 10.1038/s41389-021-00354-7

**Published:** 2021-09-22

**Authors:** Tamar Evron, Michal Caspi, Michal Kazelnik, Yarden Shor-Nareznoy, Shir Armoza-Eilat, Revital Kariv, Zohar Manber, Ran Elkon, Ella H. Sklan, Rina Rosin-Arbesfeld

**Affiliations:** 1 Department of Clinical Microbiology and Immunology, Sackler Faculty of Medicine, Tel Aviv University, Tel Aviv, Israel; 2 Department of Gastroenterology, Tel Aviv Sourasky Medical Center, Tel Aviv, Israel; 3 Department of Human Molecular Genetics and Biochemistry, Sackler Faculty of Medicine, Tel Aviv University, Tel Aviv, Israel

**Keywords:** Cancer, Cell biology

## Abstract

The Wnt signaling pathways play fundamental roles during both development and adult homeostasis. Aberrant activation of the canonical Wnt signal transduction pathway is involved in many diseases including cancer, and is especially implicated in the development and progression of colorectal cancer. Although extensively studied, new genes, mechanisms and regulatory modulators involved in Wnt signaling activation or silencing are still being discovered. Here we applied a genome-scale CRISPR-Cas9 knockout (KO) screen based on Wnt signaling induced cell survival to reveal new inhibitors of the oncogenic, canonical Wnt pathway. We have identified several potential Wnt signaling inhibitors and have characterized the effects of the initiation factor DExH-box protein 29 (DHX29) on the Wnt cascade. We show that KO of DHX29 activates the Wnt pathway leading to upregulation of the Wnt target gene cyclin-D1, while overexpression of DHX29 inhibits the pathway. Together, our data indicate that DHX29 may function as a new canonical Wnt signaling tumor suppressor and demonstrates that this screening approach can be used as a strategy for rapid identification of novel Wnt signaling modulators.

## Introduction

The Wnt signal transduction pathways, which are essential for both embryonic development and adult homeostasis, regulate numerous fundamental cell functions, including proliferation, migration, apoptosis, stem cell renewal, and differentiation [1–3]. The Wnt pathways can be broadly divided into the canonical-β-catenin dependent, and the more diverse non-canonical-β-catenin independent pathways [4]. Aberrant activation of canonical Wnt signaling is associated with a number of human diseases, including a variety of malignancies such as gastric, breast, liver, and colorectal cancer (CRC) [5].

The canonical Wnt/β-catenin pathway, similarly to other signaling cascades, is initiated at the cell membrane and its primary output involves changes in gene transcriptional programs. These changes occur by regulating the expression levels, post-translational modifications, and subcellular localization, of the Wnt signaling key effector-β-catenin [2, 6, 7]. In unstimulated cells, the Wnt-signalling cascade is silenced due to the activity of a dedicated cytoplasmic destruction complex that phosphorylates β-catenin, marking it for ubiquitination and subsequent degradation. At the core of this complex are the tumour suppressor *adenomatous polyposis coli* (APC), the scaffold protein axin, two kinases: glycogen synthase kinase-3 (GSK-3) and casein kinase 1 (CK1), and the E3-ubiquitin ligase β-TrCP [8]. Mutations in these components may lead to uncontrolled activation of the pathway and the development of cancer [5].

The Wnt-signalling cascade initiates with the binding of secreted Wnt glycoproteins to a receptor complex composed of frizzled (Fz) and low-density lipoprotein receptor-related protein 5 or 6 (LRP5/6). The binding of a Wnt ligand to the FZD-LRP5/6 complex results in recruitment of the cytoplasmic protein dishevelled, and the subsequent formation of large “signalosomes”. This process leads to disassembly of the destruction complex and stabilization of β-catenin, which translocates into the nucleus, where it associates with T-cell factor/lymphoid enhancer-binding factor (TCF/LEF) transcription factors and other components. The resultant nuclear complex upregulates Wnt target genes and is predicted to be a preferred target for novel Wnt-signalling specific therapeutic approaches [1, 2, 9, 10].

The Wnt cascade is extremely complex and is tightly regulated at different epistatic levels depending on the cellular and environmental context [11]. However, despite decades of research, crucial mechanistic gaps throughout the pathway remain to be filled, and new pathway components are still being identified [12].

To reveal unknown regulators of Wnt/β-catenin signaling, we designed and performed a genetic screen using a Genome-wide Clustered Regularly Interspaced Short Palindromic Repeats (CRISPR)/Cas9 pooled Knock-out (KO) Library, based on Wnt signaling induced cell survival. Using next-generation sequencing (NGS), we identified several new Wnt signaling inhibitors, of which, we focused on the initiation factor DExH-box protein 29 (DHX29).

## Results

### Establishment of a GeCKO screening system based on Wnt-induced cell survival under antibiotic selection

**Fig. 1:**
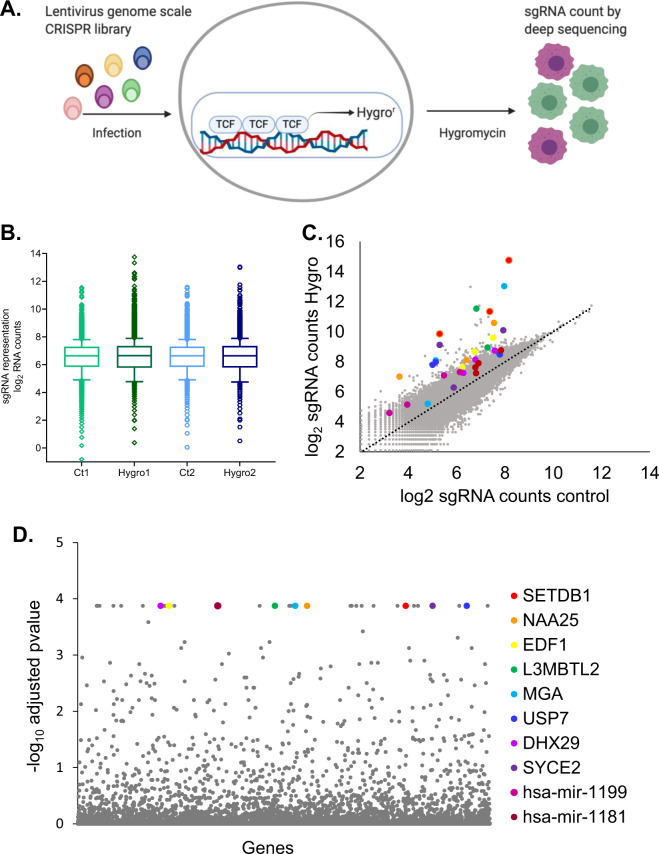
Wnt repressors CRISPR screen. **A** Schematic representation of the canonical Wnt signaling reporter plasmid and the screening strategy. **B** Tukey boxplot showing the distribution of sgRNA frequencies before and after hygromycin selection in the two biological repeats. Boxes; 25–75th percentile, whiskers; 1.5 times the interquartile range. **C** Scatter plot showing enrichment of sgRNAs targeting the top candidate genes (colored dots) compared to other sgRNAs in the library (gray dots) following hygromycin selection in the first screen repeat. **D** Scatter plot showing the significance (FDR adjusted *p*-value) of each gene in the two screen repeats. The top ten ranking genes are color coded.

The aim of the study was to use a CRISPR library in order to identify novel regulators of the canonical Wnt signaling pathway. Knocking out potential pathway repressors leads to pathway activation and subsequent hygromycin resistance of the cells mediated by a reporter plasmid TCF/HSV-TK, which we have previously used as a screening tool [13] (Fig. 1A). The reporter was stably transfected into HEK293 cells, in which the β-catenin destruction complex is active and the level of Wnt signaling activity is therefore minimal. We speculated that cells in which a Wnt inhibitor is silenced would become resistant to the hygromycin B antibiotic since the hygromycin resistance gene is regulated by the TCF-binding sites. Preliminary assays were conducted to determine hygromycin concentration and screening duration by comparing a library transduced sample with a nontransduced control sample. Following 10 days of selection with 150µgr/ml hygromycin no live cells were found in the control sample (Fig. S4). Two independent screens were conducted, followed by genomic DNA and deep sequencing analysis. Examining the sgRNA frequency distribution following hygromycin selection revealed that a subset of guide-RNAs was enriched in two independent screen repeats (Fig. 1B). Although a greater enrichment of gRNAs occurring in the cell population treated with hygromycin was expected, similar results were obtained in other screens [14, 15]. As shown in the publication of Shalem et al. longer screening periods further enrich the number of positive guides selected. Our results are comparable to the early timepoints described in these studies. Furthermore, while longer timepoints would increase the magnitude of the fold enrichment, it would probably not change the identity of the selected guides. The pooled library used contains multiple sgRNAs for each gene, and for most enriched genes, more than one sgRNA targeting the same transcript was enriched in the selected cells (Fig. 1C). Ranking the enriched genes using the MAGeCK algorithm [16] revealed a panel of novel putative Wnt repressors (Fig. 1D and Table 1). p < 2.28 × 10^−7^ for all listed genes). The observation that one of these genes is Ubiquitin Specific Peptidase 7 (USP7), which was recently identified in a different CRISPR KO screen as a potent negative regulator of Wnt signaling, further confirms our results [12]. In addition, the known Wnt signaling repressor Casein Kinase 1 Alpha 1 (CSNK1A1) was also identified in our screen [17]. The ten top-ranking genes according to the MAGeCK algorithm (from both screen repeats) are shown in Table 1.

**Table 1:** Comparison of the top hits in the two screen repeats ranked by the MAGeCK algorithm.

Gene	Screen 1	Screen 2
SETDB1	1	1
NAA25	5	2
EDF1	6	3
L3MBTL2	2	5
MGA	18	8
USP7	8	9
DHX29	14	6
SYCE2	9	10
hsa-mir-1199	3	13
hsa-mir-1181	4	19

*p* < 2.28 × 10^−7^ for all listed genes.

### Validation of the screening results

**Fig. 2:**
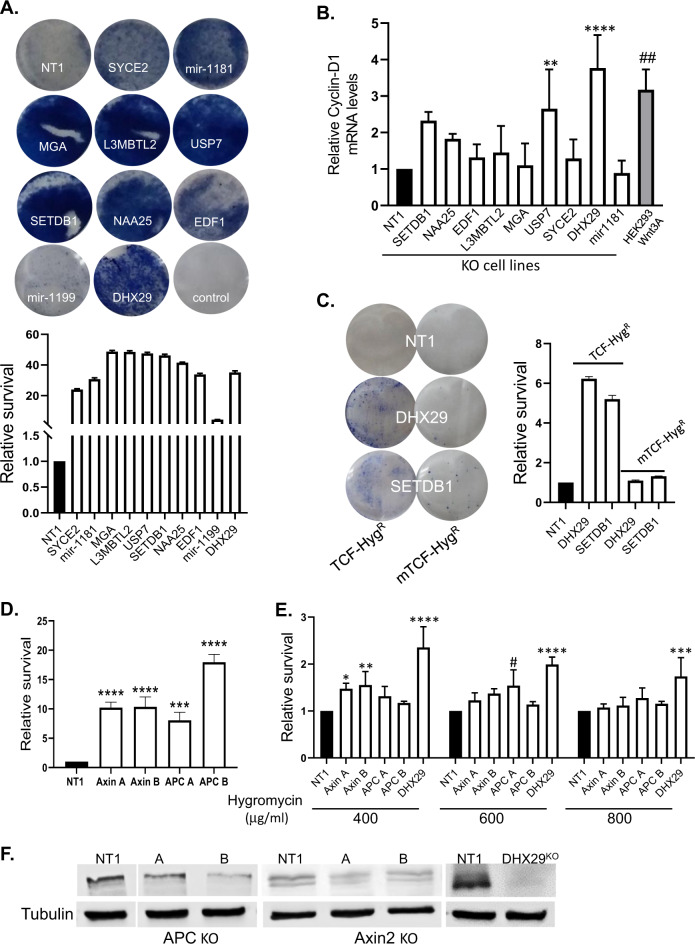
Validation of the screening results. **A** CRISPR knockout clones are resistant to hygromycin. The HEK293-TCF-hygro^r^ cell line was transduced with lentiviruses obtained from the library backbone plasmid expressing the sgRNAs targeting the top-ranking genes. Equal numbers of cells from each cell line were treated with hygromycin for 96 h, then fixed with methanol, and stained with methylene blue (upper panel). Quantification of the results shown in **A** by measurement of the absorbance at 570 nm. Results are mean ± SD from three technical repeats (Lower panel). One-way ANOVA (*P* < 0.0001) with Dunnett’s multiple comparisons test was applied, ****p < 0.0001 (for all cell lines compared to NT1 control). **B** Total RNA was extracted from HEK293 KO cell lines and used for RT-qPCR with Cyclin-D1 and actin primers. Results are mean values ± SD from 3–4 independent experiments. Ordinary one-way ANOVA (*P* < 0.0001), with Dunnett’s multiple comparisons (^††^*P* = 0.0012, **P = 0.0098, ****P < 0.0001) was applied. **C** HEK293-TCF-Hygro^r^ and HEK293-mTCF-Hygro^r^ KO cell lines were selected with 350 µgr/mL Hygromycin for 7 days, then fixed with methanol and stained with methylene blue. Quantification of the results shown by measurement of the absorbance at 570 nm. Results are mean values ± SD from 3 technical repeats. **D** HEK293-TCF-Hygro^r^ APC/Axin2 KO cell lines were selected with 400 µgr/mL Hygromycin for 7 days. PrestoBlue reagent was added to the wells and absorbance measured after 2 h incubation at 570 nm. Ordinary one-way ANOVA (*P* < 0.0001) with Dunnett’s multiple comparisons (***P = 0.0001, ****P < 0.0001) was applied. **E** HEK293-TCF-Hygro^r^ APC/Axin2, DHX29, and NT1 control KO cell lines were selected with 400/600/800 µgr/mL Hygromycin for 72 h. PrestoBlue reagent was added to the wells and fluorescence measured after 1 h incubation at 540/590 excitation/emission wavelengths. Two-way ANOVA (*P* < 0.0001), with Dunnett’s multiple comparisons (*P = 0.0320, ^†^*P* = 0.0115, **P = 0.0097, ***P = 0.0005, ****P < 0.0001) was applied. **F** Total protein from HEK293-TCF-Hygro^r^ APC/Axin2, DHX29, and NT1 control KO cell lines was harvested for western blot analysis. The blots were incubated with specific antibodies to APC, Axin2, DHX29, or tubulin.

To validate the screen results, we individually cloned sgRNAs from the top-ranking genes into the library backbone vector and established HEK293-TCF-Hygro^r^ KO stable cell lines carrying these ten specifies guides. A control cell line expressing a nontargeting sgRNA (NT1) was also prepared. The HEK293-TCF-Hygro^r^ KO cell lines were selected with hygromycin for four days, and the surviving cells were stained with methylene blue (Fig. 2A). Quantification of the methylene blue staining compared to a nontargeting control showed variable levels of survival in all cell lines. The highest levels of survival (Wnt activation) were observed with MGA, L3MBTL2, USP7, and SETDB1 (~48 fold the control value), followed by NAA25, EDF1, DHX29, and miR1181 (~35 fold the control value), SYCE2 (24 fold the control value), and miR-1199 (4 fold the control value). In order to assess the connection between the top-ranking genes and canonical Wnt signaling functionally, we measured the transcript levels of Cyclin-D1, a known Wnt target gene, by real-time qPCR analysis (Fig. 2B). Cells incubated in media containing the Wnt3A ligand were used as positive control. Elevated Cyclin-D1 mRNA levels were detected in the DHX29 and USP7 knockout (KO) cell lines (Fig. 2B). To further confirm the validity of our screening system, we mutated the TCF-binding sites in the HEK293-TCF-Hygro^r^ construct to generate the HEK293-mTCF-Hygro^r^ plasmid that was used to create stable cell lines. In these cells, as opposed to cells expressing the wild-type TCF sequences, expression of β-catenin did not result in hygromycin resistance (Supplementary Fig. 1B). We then transduced the two types of cells (wild-type TCF or mutated TCF) with DHX29 and SETDB1 (which also scored highly in our screen) gRNAs and demonstrated that hygromycin resistance is only achieved in the TCF-Hygro^r^ cells (Fig. 2C), confirming the involvement of specific TCF-mediated Wnt signaling. The absence of known Wnt regulators among our top candidates was surprising and thus we tested specific KO of APC and Axin2, two tumor suppressors which are core regulators of Wnt signaling. As our library was divided into two parts (A and B—each containing three sgRNAs for each gene), we obtained the individual APC and Axin2 gRNAs from each part and tested their ability to induce hygromycin resistance compared to the DHX29 KO. The gRNA’s effect differed between the two library parts (Fig. 2D and F), and moreover, as shown in Fig. 2E, both APC and Axin2 KO rendered a mild hygro-resistance compared to DHX29 in three different antibiotic dosages. Western blot analysis revealed that neither APC nor Axin2 KO resulted in complete elimination of their protein product. DHX29 KO, on the other hand, completely abolished protein expression and we thus continued studying its role in the Wnt signaling pathway (Fig. 2F).

### DHX29 KO does not affect cell proliferation

**Fig. 3:**
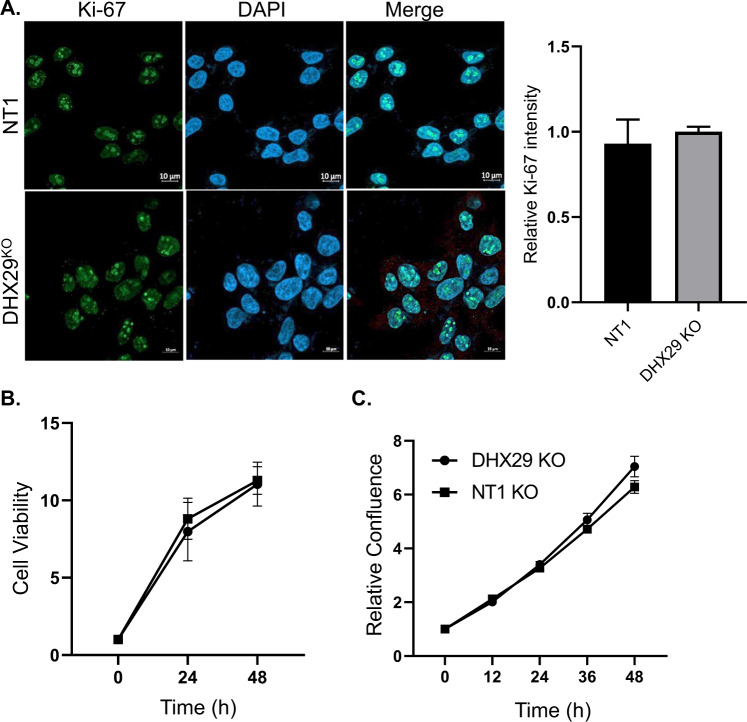
DHX29 KO does not affect cell proliferation. **A** NT1 and DHX29 knockout cells were fixed, permeabilized, and stained with an anti-Ki-67 antibody. Cell nuclei were counterstained with DAPI. Bar = 10 µm. Ki-67 staining intensity was determined by ImageJ software measuring 3 fields of 15–20 cells in each field. Results are mean values ± SD from 3 measurements. Unpaired t-test, nonsignificant. **B** HEK293-DHX29 KO and NT1 control cells were seeded in equal amounts in a 96-well plate. Cell viability was measured by adding PrestoBlue reagent and measuring absorbance according to manufacturer instructions at 24 and 48 h. (B). Alternatively, cell confluence was measured every 12 h for 2 days using IncuCyte S3 Live-Cell analysis system. Results are mean values ± SD from 3–4 repeats. Two-tailed paired *t*-test, nonsignificant.

In addition to its role as a canonical Wnt target gene, Cyclin-D1 is an important cell cycle regulatory protein that controls the transition from G1 to S phase [18]. To rule out a nonspecific effect on cell proliferation and confirm that the increased Cyclin-D1 levels observed upon DHX29 KO can be specifically attributed to Wnt signaling regulation, we tested the levels of proliferation in this cell line. Proliferation was tested using Ki-67, a proliferative marker strongly linked to cell cycle control [19]. NT1 and DHX29 KO cell lines were fixed and stained with a Ki-67 specific antibody (Fig. 3A). Immunofluorescence intensity measurements confirmed that Ki-67 staining was similar in both cell lines, indicating that there was no significant effect on cell proliferation in the DHX29 KO cells. These results were further corroborated by a proliferation assay performed with PrestoBlue, a resazurin based reagent that assesses cell viability (Fig. 3B). In addition, cell confluence was measured every 12 h for 2 days using IncuCyte S3 Live-Cell analysis system (Fig. 3C). Both methods revealed no significant differences between DHX29 KO and the NT1 control, indicating that cell survival was indeed due to Wnt-mediated hygromycin resistance.

### DHX29 represses Wnt signaling

**Fig. 4:**
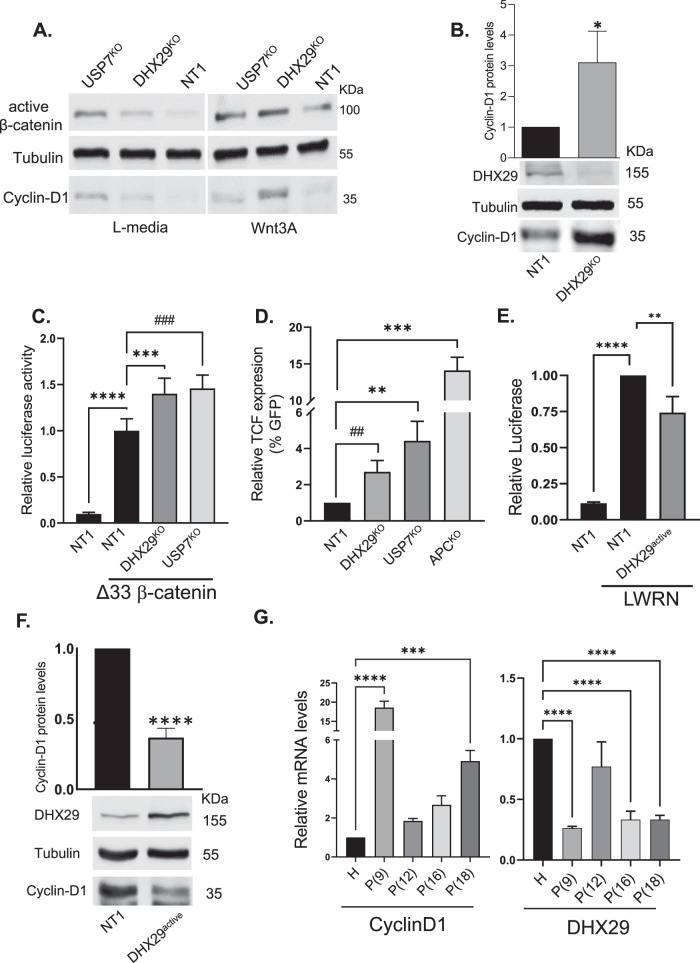
DHX29 repress Wnt signaling. **A, B** The indicated HEK293 KO cell lines were incubated with 10% L-Wnt3A or L conditioned media for 24 h. Total protein was harvested for western blot analysis. HEK293 cells transduced with a nontargeting sgRNA were used as a negative control (NT1). The blots were incubated with specific antibodies to Cyclin-D1, active β-catenin, or tubulin (**A**). The Cyclin-D1 band intensity was measured by Fusion-Capt program. Results are mean values ± SD from 3 independent experiments. Two-tailed unpaired *t*-test (*P* < 0.05), *P = 0.0234 (**B**—upper panel). **C** The indicated HEK293 KO cell lines were transfected with Renilla, TOPFLASH/FOPFLASH, and GFP or GFP- Δ33 β-catenin constructs (0.1, 0.5, and 0.5 µg per well, respectively). The cells were harvested after 48 h, and a luciferase reporter assay was performed. Results are mean values ± SD from 3–4 independent experiments. One-Way ANOVA test (*P* < 0.0001) with Dunnett’s multiple comparison test was used (***P = 0.0004, ^†††^*P* = 0.0001,****P < 0.0001). **D** The indicated HEK293 KO cell lines and HEK293-TCF-Hygro^r^ APC B KO cell line were transduced with the 7TGC lentivirus. Ninety six hours later cells were harvested for Flow Cytometry analysis. Unpaired *t*-tests (*P* < 0.05) were used (^††^*P* = 0.0089, **P = 0.0053, ***P = 0.0002). **E** DHX29-activated or control cells were transfected with Renilla and TOPFLASH/FOPFLASH constructs (0.1 and 0.5 µg per well, respectively). Fifty percent L-WRN or L C.M. was added for 24 h, after which the cells were harvested for the Luciferase assay. Results are mean values ± SD from 3 independent experiments. Ordinary One-Way ANOVA (*P* < 0.0001) with Dunnett’s multiple comparison test was used (**P = 0.0049, ****P < 0.0001). **F** Western blot analysis of DHX29-activated cells. The membranes were blotted with antibodies to DHX29, Cyclin-D1, and tubulin. The lower panel displays a representative blot. Quantification of Cyclin-D1 protein levels in the DHX29^active^ cell line. Results are mean values ± SD from 3 independent experiments. ****P < 0.0001, two-tailed unpaired *t*-test. **G** Total RNA was extracted from the indicated specimens as described in ref. [19]. RT-qPCR was performed with Cyclin-D1, DHX29, and actin primers. Results are mean values ± SD from 3–4 independent experiments. One-Way ANOVA test (*P* < 0.0001) with Dunnett’s multiple comparison test was used (***P = 0.0005, ****P < 0.0001).

Next, we measured the protein levels of unphosphorylated active β-catenin and Cyclin-D1 in the HEK293 KO cell lines, focusing on DHX29 and USP7, which was recently identified in a similar screen as a Wnt repressor [12]. Increased levels of both active β-catenin and Cyclin-D1 were observed in the two KO cell lines (Fig. 4A and B), further confirming their involvement in suppressing Wnt signaling. Interestingly, the effect of the DHX29 KO was more robust when Wnt activity was induced by the addition of the Wnt3A ligand (Fig. 4A). When the levels of DHX29 and Cyclin-D1 protein were tested in NT1 and DHX29 KO cells, the results confirmed the expected depletion of DHX29 protein in the DHX29 KO cells, accompanied by a significant increase in Cyclin-D1 protein expression by ~3 fold (Fig. 4B), similar to the increased levels of Cyclin-D1 seen when HEK293 cells were incubated with Wnt3A (Supplementary Fig. 3).

As the next step, we examined β-catenin/TCF-mediated transcription in the DHX29 and USP7 KO cell lines using the pTOPFLASH/ pFOPFLASH reporter assay. Canonical Wnt signaling was induced by β-catenin overexpression, and the signaling levels were compared to NT1 controls. As depicted in Fig. 4C, canonical Wnt signaling was significantly increased in the two cell lines confirming that DHX29 and USP7 are both Wnt signaling repressors.

In addition, we assayed β-catenin/TCF-mediated transcription using flow-cytometry analysis. NT1, DHX29, USP7, and APC (gRNAs from library B) KO cell lines were transduced with the 7TGC lentivirus, which expresses constitutive mCherry and Wnt-activation-dependent GFP (under the control of 7XTCF binding sites). Wnt signaling levels were recorded by flow-cytometry measuring the percentage of GFP expressing cells within the mCherry stained population. Canonical Wnt signaling was significantly increased in the two KO cell lines DHX29 and USP7, and in the APC positive control (Fig. 4D). This strengthens the conclusion that DHX29 and USP7 are both Wnt signaling repressors. In order to identify other proteins that may connect DHX29 to the Wnt cascade, mass spectrometry analysis of DHX29 KO compared to control cells was conducted. The analysis revealed changes in the expression of several proteins involved in Wnt signaling regulation (Table 2). On one hand, DHX29 depletion resulted in increased expression of Wnt regulated proteins, such as DHX33 and SLC7A5, or factors that transduce the Wnt signal such as ARF6, MAPK8, and UBQLN4 [20–24]. On the other hand, DHX29 silencing led to the downregulation of desmoglein-2 (DSG2), an adhesion molecule that is associated with the sequestering of β-catenin to the cell membrane and the suppression of TCF-mediated transcription [25]. Integrator complex subunit 6 (INTS6), a putative tumor suppressor shown to downregulate Wnt/β-catenin signaling was also depleted upon the elimination of DHX29 expression [26]. These results further support our hypothesis that DHX29 is involved in Wnt signaling regulation. We then examined the effects of DHX29 overexpression on canonical Wnt signaling, using CRISPR activation. One of the three sgRNAs used for CRISPR activation was the most efficient and was therefore chosen for further experiments (Supplementary Fig. 2). As expected, overexpression of DHX29 decreased canonical Wnt signaling as determined by the TOPFLASH assay (Fig. 4E), and reduced the levels of Cyclin-D1 protein (Fig. 4F). As Wnt signaling is frequently over-activated in different cancers especially colorectal cancer [5], we examined the levels of DHX29 mRNA in early adenomas (polyps) obtained from Familial Adenomatous Polyposis (FAP) patients. FAP is an autosomal-dominant colorectal cancer syndrome, caused by a germline mutation in the APC gene, characterized by hundreds of adenomatous colorectal polyps, with an almost inevitable progression to colorectal cancer at an average age of 35–40 [27]. Interestingly, DHX29 transcript levels were decreased in the polyp samples compared to the healthy tissue. The decrease was accompanied by an increase in cyclin-D1 expression levels (Fig. 4G). Taken together these results indicate that DHX29 may be involved in repressing Wnt signaling, and its absence may facilitate Wnt-mediated tumorigenesis.

**Table 2:** Mass spectrometry analysis of protein expression in the DHX29 KO cell line compared to control.

Protein symbol	Description	MASCOT score	Relation to Wnt signaling	Reference
DHX33	ATP-dependent RNA helicase	Below detection in control	Regulates β-catenin expression and is regulated by Wnt signaling	[24]
ARF6	ADP-ribosylation factor 6	28	Required for LRP6 phosphorylation and transduction of Wnt/β-catenin signaling	[21]
MAPK8	Mitogen-activated protein kinase 8	20	Promotes WNT/β-catenin signaling via phosphorylation of LRP6	[20]
SLC7A5	Large neutral amino acids transporter small subunit	17	Wnt signaling regulates amino acid transporter Slc7a5	[22]
UBQLN4	Ubiquilin-4	8	Activates Wnt/β-catenin signaling	[23]
DSG2	desmoglein-2	Below detection in DHX29 KO sample	Loss of DSG2 induced β-catenin nuclear localization and activated LEF/TCF-dependent transcription	[25]
INTS6	Integrator complex subunit 6	Below detection in DHX29 KO sample	Downregulates Wnt/β-catenin signaling and the impairment of β-catenin degradation reversed the tumor suppressor effects of INTS6	[26]

## Discussion

The CRISPR/Cas9 technology combined with genome-scale guide RNA libraries for unbiased genetic screening have been used in recent years (reviewed in refs. [28, 29]). Pooled CRISPR/Cas9 KO libraries are extremely powerful and are based on the concurrent targeting of a large number of genes in a pooled, single batch library, under conditions of one gRNA perturbation per cell (reviewed in ref. [30]). In this study, a CRISPR/Cas9 KO screen was used to identify novel canonical Wnt signaling negative regulators. Our top candidates include genes previously shown to be associated with Wnt signaling, such as USP7 [12] and SETDB1 [31]. In addition, proteins not previously known to affect Wnt signaling, such as DHX29, were also identified. It should be noted that different types of genome-wide screens (gene-trap, siRNA and CRISPR) aimed at the identification of new Wnt signaling components yield different hits [32, 33]. Importantly, even the most known and robust Wnt signaling regulators were not highly scored in most cases and the top hits widely differ between individual screens [12, 32–40]. This observation was previously discussed [32] and it was suggested that distinct screening systems i.e., different libraries or cell lines used, differences in phenotypic scoring, the length of the screen and other factors affecting knockout efficiency influence the screen results. The different screens may complement each other and lead to the identification of a larger variety of direct and indirect Wnt signaling modulators. It was thus proposed that the canonical Wnt/β-catenin pathway consists of several conserved core components and has additional context-dependent modulators [32]. APC, for example, may be challenging for CRISPR targeting as it is a very large protein, which might be difficult to target by a single sgRNA (as designed by the MOI of our screen). In addition, APC is a stable protein which might remain in cells for long periods of time. Moreover, complete loss of APC function is rarely found even in cancer cells which harbor APC mutations. This is usually explained by the “Just right” model stating that the impairment of APC function in cancer allows sufficient accumulation of β-catenin that facilitates tumor formation [13], as opposed to complete loss of APC function that leads to excessive β-catenin accumulation triggering apoptosis [14]. We thus speculate that knockout of genes such as APC, if not lethal, most likely renders the cells more fragile and thus less susceptible to acquire antibiotic resistance that would allow them to sustain the prolonged hygromycin selection (unlike the GFP reporter screen used by Ji et al.). The fact that CSNKIA1 was the only known core Wnt signaling component that scored highly in our screen raises the possibility that this type of screen is predisposed towards isolating moderate Wnt effectors.

The DEAD/H box RNA helicase 29—DHX29, which has not been previously connected with the Wnt pathway, is a ubiquitously expressed protein associated with the 40S ribosomal subunit [41]. DHX29 is an initiation factor contributing to start codon selection [42], and is required for efficient 48S complex formation on mRNAs with highly structured 5′-UTRs [43]. DHX29 silencing was shown to inhibit cancer cell proliferation [41] and it is significantly overexpressed in non-small cell lung carcinoma‏ (NSCLC) malignancies [44]. However, as DHX29 may harbor different mechanisms of action, and has an important role in initiation codon selection [42], it may also possess anticancer properties. It was suggested that DHX29 carries additional roles beyond translation, such as recognizing double-stranded RNA and specific interaction with melanoma differentiation-associated protein 5 (MDA5) to enhance innate antiviral immunity [45]. Interestingly, other DEAD/H box RNA helicases have also been shown to affect Wnt signaling; DDX3 was shown to be a regulatory subunit of CK1 that regulates canonical Wnt signaling [46, 47], and DDX3 knockdown was recently shown to inhibit AKT activity and Wnt signaling [48]. DHX15 has been shown to mediate Wnt-induced antimicrobial protein expression in specific colonic cells [49]. DDX5 has also been implicated in the regulation of Wnt signaling [50], and our current study suggests that another RNA helicase, DHX29, affects Wnt/β-catenin signaling. Translational control plays an important role in cell growth and tumorigenesis and is tightly regulated during development. Here we demonstrate that DHX29 mRNA levels are reduced in adenomas obtained from FAP patients. However, a previous study has shown that DHX29 is overexpressed in various types of cancers, including glioblastoma multiforme, metastatic melanoma, ovarian endometrioid carcinoma and ovarian serous adenocarcinoma [41]. This discrepancy may be explained by the stage of malignancy of the different samples. It is possible that DHX29 is involved in the translational initiation of different sets of genes in different stages of cancer development. It is further demonstrated that DHX29 promotes tumorigenesis in some experimental setups. However, targeting DHX29 in order to suppress cancer cell growth in a clinical setup may prove difficult due to the complexity of its function during cancer development and progression.

Our genome-wide screen has identified a number of putative canonical Wnt signaling inhibitors. Future studies are needed to reveal the exact mechanisms and roles of these candidates in regulating the Wnt pathway and the way that they could be harnessed, in the future, to combat Wnt signaling-related disorders.

## Materials and methods

### Cell culture and transfections

HEK293, L (ATCC CRL-2648), L Wnt-3A (ATCC CRL-2647), and L-WRN cells (ATCC CRL-3276) were grown in Dulbecco’s modified Eagle’s medium (DMEM, Biological Industries) supplemented with 10% fetal bovine serum (GIBCO) and 1% penicillin-streptomycin (Biological Industries). L Wnt-3A cells were grown in presence of G418 (0.4 mg/mL, EMD Millipore). L-WRN cells were grown in the presence of 0.5 mg/mL G418 and 0.5 mg/mL Hygromycin B (Invivogen). Conditioned medium was prepared from L, L Wnt-3A, and L-WRN cell lines according to product specifications.

HEK293FT cells were grown in D10 medium; DMEM with 10% fetal calf serum, 1% penicillin-streptomycin, 1% L-Glutamine, 1% Sodium Pyruvate, 1% Sodium Bicarbonate (Biological Industries, Beit-Haemek, Israel), with 0.5 mg/mL G418 [51]. Transfections were performed with jetPEI (Polyplus Transfection) following the manufacturer’s protocols.

### Plasmids

The Wnt signaling reporter plasmid TCF/HSV-TK has been described previously [52]. In this work, Puromycin was replaced with Hygromycin B coding region downstream to the three consensus TCF-binding sites, using HindIII and HpaI restriction enzymes. Mutated TCF/HSV-TK was prepared by introducing mutated binding sites to the TCF/HSV-TK reporter plasmid using PciI and BamHI restriction enzymes. The Wnt-responsive TCF-dependent luciferase constructs pTOPFLASH (containing multi TCF-binding sites linked to a luciferase reporter) and its mutated version pFOPFLASH were kindly provided by Prof. H. Clevers (Center for Biomedical Genetics, Hubrecht, The Netherlands) [53]. The pCMV-Renilla expression plasmid, used to evaluate transfection efficiency, was purchased from Promega (Madison, WI, USA). 7xTcf-eGFP // SV40-mCherry lentiviral vector (7TGC) was a gift from Roel Nusse (Addgene plasmid # 24304).

GFP-β-catenin was constructed by inserting the β-catenin cDNA into pEGFP-C1 (Clontech) using *Bam*HI and *Xba*I restriction sites. This expression vector was used as a template to replace serine 33 with alanine to generate GFP-S33A-β-catenin by using the QuikChange site-directed mutagenesis kit (Stratagene).

### HEK293-TCF-Hygro^r^ stable cell line

The TCF/HSV-TK reporter plasmid was linearized using PciI (to avoid damaging the reporter sites) and transfected into HEK293 cells. Cells stably expressing the reporter were selected with 0.85 mg/mL G418, and the obtained colonies were isolated and expanded. Colonies were tested for inducible hygromycin resistance by transfection with 3 μg of the GFP-S33A-β-catenin (Δ33 β-catenin-GFP), or a GFP only vector. Following transfection, hygromycin (0.8 mg/mL) was added for 72 h, and the cells were then fixed with methanol and stained with 0.1% methylene blue (Sigma). The colony with the highest level of hygromycin resistance (Fig. S1A, designated HEK293-TCF-Hygro^r^) was used for the CRISPR screen.

### sgRNA library amplification and lentivirus production

The Human CRISPR knockout pooled library (GeCKO v2) was a gift from Prof. Feng Zhang (Addgene, 1000000048). The library is divided to two parts (A and B), each containing three independent gRNAs for 19,050individual protein-coding genes, four independent gRNAs targeting miRNAs, and 1000 control nontargeting guides. The library was amplified according to the depositor’s protocol [54]. For lentivirus production, HEK293FT cells were plated at ~50% confluence in six 15 cm plates. The next day, the cells were transfected with the lentivirus packaging plasmids pLP1 (7.8 µg), pLP2 (3 µg), VSV-G (4.2 µg), and the amplified library (12 µg) per plate, using jetPEI. The supernatant was harvested 40 h post-transfection, filtered, and stored at −80 °C [51]. The library titer was determined by transduction in HEK293 cells using the Presto Blue cell viability reagent (Thermo), as described previously [54].

### CRISPR knockout (KO) screen

Prior to screening initiation: Stable HEK293 cells expressing the reporter plasmid (TCF-Hygro^r^), were screened for inducible hygromycin resistance following activation of the Wnt pathway by overexpressing a constitutively active β-catenin mutant (Δ33 β-catenin) that cannot be phosphorylated by GSK-3β and is therefore not susceptible to proteasomal degradation [55]. The clone with the highest degree of inducible hygromycin resistance (Fig. S1) was selected for the CRISPR screen.

Screening strategy: for transduction with the lentiviruses carrying the library sgRNAs, HEK293-TCF-Hygro^r^ cells were seeded into 12-well plates at a density of 3 × 10^6^ cells per well. A total of 1.65 × 10^8^ HEK293-TCF-Hygro^r^ cells were transduced at a MOI of 0.2 to yield 3.3 × 10^7^ transduced cells. Since the library (part A) contains 65,384 sgRNA constructs, this number is sufficient for the transduction of 500 cells with each unique sgRNA [54]. Untransduced cells were used as a control. The cells were spinoculated at 1000 × *g* for 2 h at 25 °C. After 24 h, the cells were pooled and replated in 15 cm plates at 6 × 10^6^ cells per plate. The cells were then selected with 0.5 µg/mL puromycin (Fermentek) for 7 days. Untransduced control cells were selected in a similar manner. After seven days of selection, the untransduced control plates did not contain any viable cells. Surviving cells expressing sgRNAs were pooled, and 4 × 10^7^ cells were pelleted and kept as an untreated control at −20 °C. The remaining cells were replated in 15 cm plates at 6 × 10^6^ cells per plate and selected with 150 µg/mL hygromycin B for 10 days. During the incubation period, the medium and antibiotics were replaced every 3 days. Following treatment, 4 × 10^7^ cells were collected for further analysis. The screen was performed twice. Genomic DNA was extracted from all samples (two untreated controls and two independent screened samples) as described previously [56]. sgRNA sequences were amplified from the genomic DNA for NGS using PCR with primers containing Illumina adaptors, complementary sequences to the sgRNA vector, and a unique barcode sequence for each sample, to enable multiplexed analysis [54]. The pooled samples from both screens were sequenced using the Illumina NextSeq platform (Crown Institute for Genomics, Weizmann Institute of Science). The results were analyzed using MAGeCK [16, 57] with default parameters as described previously [54]. Raw sgRNA sequencing read numbers, the MAGeCK scored and ranked list of genes and the Count summary from both screen repeats are provided in the supplementary materials (Supplementary Files S1–S3).

### Validation of screen hits

To validate the screen results, sgRNAs for the top-ranking genes were individually cloned into the library backbone plasmid (Addgene, 52961). sgRNAs with nontargeting sequences (NT1) were used as controls. Lentiviruses produced from these plasmids were used to transduce HEK293-TCF-Hygro^r^ cells or the parental HEK293 cells. The cells were selected with 0.5 μg/mL puromycin for 7 days to create stable cell lines. HEK293-TCF-Hygro^r^ knockout cell lines were selected with hygromycin for 4 days, and the surviving cells were fixed with methanol and stained with 0.1% methylene blue (Sigma). To quantify the methylene blue staining the dye was extracted with 500 µL elution buffer (50% ethanol, 49% phosphate buffered saline, and 1% acetic acid). The eluted samples were loaded in triplicates to a 96-well plate, 100 µL per well, and absorbance at 570 nm was measured using a plate reader.

### Cell viability assays

PrestoBlue viability reagent (Thermofisher, Ref A13261) was used according to the manufacturer’s protocol. Measurement of absorbance/fluorescence at 570 nm and 540/590 (excitation/emission), respectively, using Epoch microplate Spectrophotometer (BioTek).

IncuCyte S3 Live-Cell analysis system was used to measure cell confluence at the SICF, Tel Aviv University.

### CRISPR activation

CRISPR activation uses sgRNAs directed to various regions in endogenous promoters to recruit a catalytically inactive Cas9 (dCas9) fused to a transcription activator and consistently induces high gene expression levels [14, 58–64]. Three different sgRNA sequences targeting the DHX29 promoter obtained from the lentiSAMv2 library sequences (Addgene, 1000000078) were cloned into the lentiSAMv2 library backbone (Addgene, 75112) and packaged into lentiviral particles. sgRNAs with nontargeting sequences (NT1) were used as controls. HEK293 cells stably expressing the activation domain construct MS2-P65-HSF1 (Addgene, 89308, termed HEK293-active) were transduced with these lentiviral particles. These were selected with 5µg/mL Blasticidin (Invivogen) for 7 days to generate three distinct DHX29-activated cell lines termed DHX29^Active^ #1/2/3 or NT1^Active^ as control.

### HEK293-TCF-Hygro^r^ and HEK293-mTCF-Hygro^r^ cell lines

The TCF/HSV-TK or mutated TCF/HSV-TK (mTCF/HSV-TK) plasmids were transfected to HEK293 cells. Cells stably expressing the reporters (designated HEK293-TCF-Hygro^r^ or HEK293-mTCF-Hygro^r^) were selected with 0.95 mg/mL G418. Inducible Hygromcyin resistance was tested in the cell lines by transfection with 3 μg of the Δ33 β-catenin-GFP, or a GFP only vector. Following transfection, hygromycin (0.4 mg/mL) was added for 72 h, and the cells were then fixed with methanol and stained with methylene blue (Fig. S1B).

To validate the specificity of the screen, HEK293-TCF-Hygro^r^ or HEK293-mTCF-Hygro^r^ were transduced with the KO constructs targeting the top-ranking genes SETDB1, DHX29 and NT1 as control (sgRNAs individually cloned into the library backbone plasmid (Addgene, 52961). The cells were selected with 0.5 μg/mL puromycin for seven days to create stable cell lines. The HEK293-TCF/mTCF-Hygro^r^ KO cell lines were selected with 350 µgr/mL Hygromycin for 7 days and the surviving cells were fixed with methanol and stained with 0.1% methylene blue (Sigma). To quantify the methylene blue staining the dye was extracted as described above, and absorbance at 570 nm was measured as described above using a plate reader.

### HEK293-TCF-Hygro^r^ APC and Axin2 KO cell lines

Sequences of sgRNAs targeting APC and Axin2 (library A and B) were taken from the GeCKO library data (Addgene 1000000048). These were individually cloned into the library backbone plasmid (Addgene, 52961). Each library (A or B) contains three sgRNAs per gene, which were pooled together and packaged to lentiviral constructs creating two lentiviral pooled constructs per gene, A and B. HEK293-TCF-Hygro^r^ cells were transduced with the APC or Axin2 Library A and B constructs, and selected with 0.5 μg/mL puromycin for 7 days to create stable cell lines.

### Luciferase reporter assay

Cells were seeded at 1 × 10^5^ cells per well in a 24-well plate 24 h before transfection. Cells were transfected with pTopflash/pFopflash, pEGFP-β-catenin/pEGFP-C1, and SV40-Renilla plasmids, using jetPEI. Following transfection (48 h), the cells were harvested and subjected to luciferase assay according to the manufacturer’s instructions. In all assays, Topflash activity was normalized to Fopflash activity. Transfection efficiency was normalized using a Renilla luminescence control.

### Flow cytometry

The cells were washed with PBS and collected in 300 μl buffer (1% fetal bovine serum, 5 mmol/L EDTA, and 0.1% sodium azide in PBS). Events were acquired using CytoFLEX flow cytometer and analyzed using Kaluza Flow Cytometry Software. Gating strategy was based on live singlets, percentage of GFP+ cells was determined out of pregated mCherry+ cells, based on matching untransfected (unstained) samples.

### Immunofluorescence

HEK293-DHX29 or NT1 knockout cells were grown on coverslips and fixed with 4% paraformaldehyde for 20 min. After three PBS washes, the fixed cells were permeabilized with 0.1% Triton X-100 for 10 min and blocked with bovine serum albumin for 1 h. Cells were then incubated at room temperature for 1 h with the primary antibodies and then following three PBS washes were incubated for an additional hour with the secondary antibody. Cell nuclei were stained with 4–6′ diamidino-2 phenylindole (DAPI, Sigma). Cells were visualized using a confocal laser microscope (LSM800, Carl Zeiss).

### Western blot analysis

Total protein was harvested from cells with lysis buffer (100 mM NaCl, 50 mM Tris, pH 7.5, 1% Triton X-100, 2 mM EDTA) containing protease inhibitor cocktail (Sigma). Extracts were clarified by centrifugation at 12,000 × *g* for 15 min at 4 °C. Following SDS–polyacrylamide gel electrophoresis, proteins were transferred to nitrocellulose membranes and blocked with 5% low-fat milk. Membranes were incubated with primary antibodies overnight, washed with PBS containing 0.001% Tween-20 (PBST), and incubated with the appropriate horseradish peroxidase-conjugated secondary antibody for 1 h. After washing in PBST, membranes were subjected to enhanced chemiluminescence detection analysis. Band intensity was quantified using the Fusion-Capt program.

### Antibodies

The following antibodies were used: rabbit anti-non-phospho (Active) β-catenin (Cell signaling, 19807, 1:2000), mouse anti-tubulin (Sigma, T-9026, 1:10,000), mouse anti-Cyclin-D1 (Santa Cruz Biotechnology sc-20044, 1:500), mouse anti-DHX29 (Santa Cruz Biotechnology sc-81080, 1:200), rabbit anti-Ki-67 (Abcam, ab15580WB, immunofluorescence 1:250), mouse anti-APC (Calbiochem, OP44), rabbit anti-Axin2 (Cell Signaling, 76G6, 1:1000). The secondary antibodies used were HRP anti-mouse and anti-rabbit (Jackson Immuno Research, 1:10 000) or Alexa fluor 488 goat anti-rabbit 1:500 (Invitrogen, A11034).

### Real-time qPCR

Total RNA was isolated from the cultured cells using TRI reagent (Bio-lab) and an RNA extraction kit (ZYMO) according to the manufacturer’s protocol. Total RNA (1 μg) was reverse transcribed using the iScript cDNA Synthesis Kit (Bio-Rad) according to the manufacturer’s instructions. Real-time PCR was performed using the CFX Connect Real-Time PCR Detection System (Bio-Rad) using a SYBR Green Master mix (PCR Biosystems). β-actin was used as a housekeeping control. The primers for the amplification of the specific cDNA sequences were:

Cyclin D1 Fw: 5’CTGTGCATCTACACCGACAA3’

Cyclin D1 Rv: 5’CTTGAGCTTGTTCACCAGGA3’

DHX29 Fw: 5’GGGAGCTACTTTAGCCCTTTACC3’

DHX29 Rv: 5’CTCCAGCCAAACATCTCGGT3’

β-actin Fw: 5′CCTGGCACCCAGCACAAT3′

β-actin Rv: 5′GGGCCGGACTCGTCATACT3′

### FAP patient Biopsy samples

Adenoma and healthy surrounding tissue samples were collected in liquid nitrogen. RNA was extracted using the AllPrep DNA/RNA/protein kit (QIAGEN) following manufacture’s protocol. The samples were obtained for a previous study [19], which was approved by the local IRB committee and registered at the NIH website (NCT02175914). All patients or their legal guardians signed informed consent forms prior to study enrollment.

### Mass spectrometry analysis

Equal amounts of HEK293-DHX29 or NT1 knockout cells were pelleted and sent for Mass spectrometry analysis at the Smoler proteomics center, Israel Institute of Technology. The samples were digested by trypsin, analyzed by LC-MS/MS on Q-Exactive (Thermo), and identified by Discoverer software against a Human database. The results were semi-quantified by calculating the peak area of each peptide as the average of the three most intense peptides from each protein. The area scores of the protein profile from the HEK293-DHX29 KO sample were compared to that in the NT1 control to identify up and downregulated proteins.

### Statistical methods

Data were analyzed using Graphpad Prism software (version 9.0, GraphPad, La Jolla, CA) and the results are presented as the mean with standard deviation of 3–5 repeats. An unpaired *t*-test or analysis of variance (ANOVA) to assess the significance of variations; multiple comparisons were conducted according to software recommendations.

## Supplementary information

SUPP. LEGENDS

supp. figs

supp. file 1

supp. file 2

supp. file 3

## References

[1] Flanagan DJ, Vincan E, Phesse TJ (2019). Wnt signaling in cancer: not a binary ON:OFF switch. Cancer Res.

[2] Nusse R, Clevers H (2017). Wnt/beta-catenin signaling, disease, and emerging therapeutic modalities. Cell.

[3] Steinhart Z, Angers S. Wnt signaling in development and tissue homeostasis. Development. 2018;145:1–8. 10.1242/dev.146589 29884654

[4] Gordon MD, Nusse R (2006). Wnt signaling: multiple pathways, multiple receptors, and multiple transcription factors. J Biol Chem.

[5] Zhan T, Rindtorff N, Boutros M (2017). Wnt signaling in cancer. Oncogene.

[6] Hanley MP, Hahn MA, Li AX, Wu X, Lin J, Wang J (2017). Genome-wide DNA methylation profiling reveals cancer-associated changes within early colonic neoplasia. Oncogene.

[7] Stamos JL, Chu ML, Enos MD, Shah N, Weis WI (2014). Structural basis of GSK-3 inhibition by N-terminal phosphorylation and by the Wnt receptor LRP6. Elife.

[8] Stamos JL, Weis WI (2013). The beta-catenin destruction complex. Cold Spring Harb Perspect Biol.

[9] Angers S, Moon RT (2009). Proximal events in Wnt signal transduction. Nat Rev Mol Cell Biol.

[10] Caspi M, Wittenstein A, Kazelnik M, Shor-Nareznoy Y, Rosin-Arbesfeld R (2021). Therapeutic targeting of the oncogenic Wnt signaling pathway for treating colorectal cancer and other colonic disorders. Adv Drug Deliv Rev.

[11] Wiese KE, Nusse R, van Amerongen R. Wnt signalling: conquering complexity. Development. 2018;145:1–9. 10.1242/dev.165902 29945986

[12] Ji L, Lu B, Zamponi R, Charlat O, Aversa R, Yang Z (2019). USP7 inhibits Wnt/beta-catenin signaling through promoting stabilization of Axin. Nat Commun.

[13] Skalka N, Caspi M, Caspi E, Loh YP, Rosin-Arbesfeld R, Carboxypeptidase E (2013). a negative regulator of the canonical Wnt signaling pathway. Oncogene.

[14] Dukhovny A, Lamkiewicz K, Chen Q, Fricke M, Jabrane-Ferrat N, et al. A CRISPR activation screen identifies genes that protect against zika virus infection. J Virol. 2019;93:211–19. 10.1128/JVI.00211-19 PMC6675891 31142663

[15] Shalem O, Sanjana NE, Hartenian E, Shi X, Scott DA, Mikkelson T (2014). Genome-scale CRISPR-Cas9 knockout screening in human cells. Science.

[16] Li W, Xu H, Xiao T, Cong L, Love MI, Zhang F (2014). MAGeCK enables robust identification of essential genes from genome-scale CRISPR/Cas9 knockout screens. Genome Biol.

[17] MacDonald BT, Tamai K, He X (2009). Wnt/beta-catenin signaling: components, mechanisms, and diseases. Dev Cell.

[18] Xie M, Zhao F, Zou X, Jin S, Xiong S (2017). The association between CCND1 G870A polymorphism and colorectal cancer risk: a meta-analysis. Medicine (Baltimore).

[19] Kariv R, Caspi M, Fliss-Isakov N, Shorer Y, Shor Y, Rosner G (2020). Resorting the function of the colorectal cancer gatekeeper adenomatous polyposis coli. Int J Cancer.

[20] Červenka I, Wolf J, Mašek J, Krejci P, Wilcox WR, Kozubík A (2011). Mitogen-activated protein kinases promote WNT/beta-catenin signaling via phosphorylation of LRP6. Mol Cell Biol.

[21] Kim W, Kim SY, Kim T, Kim M, Bae DJ, Choi HI (2013). ADP-ribosylation factors 1 and 6 regulate Wnt/beta-catenin signaling via control of LRP6 phosphorylation. Oncogene.

[22] Poncet N, Halley PA, Lipina C, Gierliński M, Dady A, Singer GA (2020). Wnt regulates amino acid transporter Slc7a5 and so constrains the integrated stress response in mouse embryos. EMBO Rep.

[23] Yu Y, Xu P, Cui G, Xu X, Li K, Chen X (2020). UBQLN4 promotes progression of HCC via activating wnt-beta-catenin pathway and is regulated by miR-370. Cancer Cell Int.

[24] Zhu Y, Du Y, Zhang Y (2020). DHX33 promotes colon cancer development downstream of Wnt signaling. Gene.

[25] Park J, Son Y, Lee NG, Lee K, Lee DG, Song J (2018). DSG2 is a functional cell surface marker for identification and isolation of human pluripotent stem cells. Stem Cell Rep.

[26] Chen H, Shen HX, Lin YW, Mao YQ, Liu B, Xie LP (2018). Small RNA-induced INTS6 gene up-regulation suppresses castration-resistant prostate cancer cells by regulating beta-catenin signaling. Cell Cycle.

[27] Galiatsatos P, Foulkes WD (2006). Familial adenomatous polyposis. Am J Gastroenterol.

[28] Shalem O, Sanjana NE, Zhang F (2015). High-throughput functional genomics using CRISPR-Cas9. Nat Rev Genet.

[29] Miles LA, Garippa RJ, Poirier JT (2016). Design, execution, and analysis of pooled in vitro CRISPR/Cas9 screens. FEBS J.

[30] Schuster A, Erasimus H, Fritah S, Nazarov PV, van Dyck E, Niclou SP (2019). RNAi/CRISPR screens: from a Pool to a Valid Hit. Trends Biotechnol.

[31] Sun QY, Ding LW, Xiao JF, Chien W, Lim SL, Hattori N (2015). SETDB1 accelerates tumourigenesis by regulating the WNT signalling pathway. J Pathol.

[32] Major MB, Roberts BS, Berndt JD, Marine S, Anastas J, Chung N (2008). New regulators of Wnt/beta-catenin signaling revealed by integrative molecular screening. Sci Signal.

[33] Wan C, Mahara S, Sun C, Doan A, Chua HK, et al. Genome-scale CRISPR-Cas9 screen of Wnt/β-catenin signaling identifies therapeutic targets for colorectal cancer. Sci Adv. 2021;7. PMID: 34138730. 10.1126/sciadv.abf2567 PMC8133758 34138730

[34] Callow MG, Tran H, Phu L, Lau T, Lee J, Sandoval WN (2011). Ubiquitin ligase RNF146 regulates tankyrase and Axin to promote Wnt signaling. PLoS ONE.

[35] DasGupta R, Kaykas A, Moon RT, Perrimon N (2005). Functional genomic analysis of the Wnt-wingless signaling pathway. Science.

[36] Firestein R, Bass AJ, Kim SY, Dunn IF, Silver SJ, Guney I (2008). CDK8 is a colorectal cancer oncogene that regulates beta-catenin activity. Nature.

[37] Lebensohn AM, Dubey R, Neitzel LR, Tacchelly-Benites O, Yang E, et al. Comparative genetic screens in human cells reveal new regulatory mechanisms in WNT signaling. Elife. 2016;5:1–40. 10.7554/eLife.21459 PMC5257257 27996937

[38] Murakami K, Terakado Y, Saito K, Jomen Y, Takeda H, Oshima M (2021). A genome-scale CRISPR screen reveals factors regulating Wnt-dependent renewal of mouse gastric epithelial cells. Proc Natl Acad Sci USA.

[39] Steinhart Z, Pavlovic Z, Chandrashekhar M, Hart T, Wang X, Zhang X (2017). Genome-wide CRISPR screens reveal a Wnt-FZD5 signaling circuit as a druggable vulnerability of RNF43-mutant pancreatic tumors. Nat Med.

[40] Tang W, Dodge M, Gundapaneni D, Michnoff C, Roth M, Lum L (2008). A genome-wide RNAi screen for Wnt/beta-catenin pathway components identifies unexpected roles for TCF transcription factors in cancer. Proc Natl Acad Sci USA.

[41] Parsyan A, Shahbazian D, Martineau Y, Petroulakis E, Alain T, Larsson O (2009). The helicase protein DHX29 promotes translation initiation, cell proliferation, and tumorigenesis. Proc Natl Acad Sci USA.

[42] Pisareva VP, Pisarev AV (2016). DHX29 reduces leaky scanning through an upstream AUG codon regardless of its nucleotide context. Nucleic Acids Res.

[43] Pisareva VP, Pisarev AV, Komar AA, Hellen CU, Pestova TV (2008). Translation initiation on mammalian mRNAs with structured 5’UTRs requires DExH-box protein DHX29. Cell.

[44] Attar-Schneider O, Drucker L, Gottfried M (2016). Migration and epithelial-to-mesenchymal transition of lung cancer can be targeted via translation initiation factors eIF4E and eIF4GI. Lab Invest.

[45] Zhu Q, Tan P, Li Y, Lin M, Li C, Mao J (2018). DHX29 functions as an RNA co-sensor for MDA5-mediated EMCV-specific antiviral immunity. PLoS Pathog.

[46] Cruciat CM, Dolde C, de Groot RE, Ohkawara B, Reinhard C, Korswagen HC (2013). RNA helicase DDX3 is a regulatory subunit of casein kinase 1 in Wnt-beta-catenin signaling. Science.

[47] Yang F, Fang E, Mei H, Chen Y, Li H, Li D (2019). Cis-acting circ-CTNNB1 promotes beta-catenin signaling and cancer progression via DDX3-mediated transactivation of YY1. Cancer Res.

[48] Perfetto M, Xu X, Lu C, Shi Y, Yousaf N, et al. The RNA helicase DDX3 induces neural crest by promoting AKT activity. Development. 2021;148:PMC7847268. 10.1242/dev.184341 PMC7847268 33318149

[49] Wang Y, He K, Sheng B, Lei X, Tao W, et al. The RNA helicase Dhx15 mediates Wnt-induced antimicrobial protein expression in Paneth cells. Proc Natl Acad Sci USA. 2021;118. 10.1073/pnas.2111936118. 10.1073/pnas.2017432118 PMC7848544 33483420

[50] Zhang M, Weng W, Zhang Q, Wu Y, Ni S, Tan C (2018). The lncRNA NEAT1 activates Wnt/beta-catenin signaling and promotes colorectal cancer progression via interacting with DDX5. J Hematol Oncol.

[51] Cohen J, Raviv S, Adir O, Padmanabhan K, Soffer A, Luxenburg C (2019). The Wave complex controls epidermal morphogenesis and proliferation by suppressing Wnt-Sox9 signaling. J Cell Biol.

[52] Caspi E, Rosin-Arbesfeld R (2008). A novel functional screen in human cells identifies MOCA as a negative regulator of Wnt signaling. Mol Biol Cell.

[53] Morin PJ, Sparks AB, Korinek V, Barker N, Clevers H, Vogelstein B (1997). Activation of beta-catenin-Tcf signaling in colon cancer by mutations in beta-catenin or APC. Science.

[54] Joung J, Konermann S, Gootenberg JS, Abudayyeh OO, Platt RJ, Brigham MD (2017). Genome-scale CRISPR-Cas9 knockout and transcriptional activation screening. Nat Protoc.

[55] Golan T, Yaniv A, Bafico A, Liu G, Gazit A (2004). The human Frizzled 6 (HFz6) acts as a negative regulator of the canonical Wnt. beta-catenin signaling cascade. J Biol Chem.

[56] Chen S, Sanjana NE, Zheng K, Shalem O, Lee K, Shi X (2015). Genome-wide CRISPR screen in a mouse model of tumor growth and metastasis. Cell.

[57] Wang B, Wang M, Zhang W, Xiao T, Chen CH, Wu A (2019). Integrative analysis of pooled CRISPR genetic screens using MAGeCKFlute. Nat Protoc.

[58] Chavez A, Scheiman J, Vora S, Pruitt BW, Tuttle M, P R Iyer E (2015). Highly efficient Cas9-mediated transcriptional programming. Nat Methods.

[59] Gilbert LA, Horlbeck MA, Adamson B, Villalta JE, Chen Y, Whitehead EH (2014). Genome-scale CRISPR-Mediated control of gene repression and activation. Cell.

[60] Konermann S, Brigham MD, Trevino AE, Joung J, Abudayyeh OO, Barcena C (2015). Genome-scale transcriptional activation by an engineered CRISPR-Cas9 complex. Nature.

[61] Maeder ML, Linder SJ, Cascio VM, Fu Y, Ho QH, Joung JK (2013). CRISPR RNA-guided activation of endogenous human genes. Nat Methods.

[62] Mali P, Aach J, Stranges PB, Esvelt KM, Moosburner M, Kosuri S (2013). CAS9 transcriptional activators for target specificity screening and paired nickases for cooperative genome engineering. Nat Biotechnol.

[63] Perez-Pinera P, Kocak DD, Vockley CM, Adler AF, Kabadi AM, Polstein LR (2013). RNA-guided gene activation by CRISPR-Cas9-based transcription factors. Nat Methods.

[64] Tanenbaum ME, Gilbert LA, Qi LS, Weissman JS, Vale RD (2014). A protein-tagging system for signal amplification in gene expression and fluorescence imaging. Cell.

