# Supplementary material for: A CRISPR knockout screen reveals new regulators of canonical Wnt signaling
Source: Oncogenesis. 2021 Sep 22;10(9):63. doi: 10.1038/s41389-021-00354-7 (PMC8458386; doi:10.1038/s41389-021-00354-7)
Supplement: Supplementary file 1 — SUPP. LEGENDS [file 41389_2021_354_MOESM1_ESM.docx]

**Supplementary legends**

**Supplementary Figure 1.**  **A.** HEK293-TCF-Hygro^r^ cell lines were transfected with Δ33 β-catenin-GFP or a GFP only vector as a control. Hygromycin (0.8 mg/mL) was added for 72h. The cells were then fixed and stained with methylene blue. **B.** HEK293-TCF/mTCF-Hygro^r^ and HEK293-TCF-Hygro^r^ cell line #42 (used for the CRISPR screen) were transfected with Δ33 β-catenin-GFP or a GFP only vector as a control. Hygromycin (0.4 mg/mL) was added for 72h. The cells were then fixed and stained with methylene blue.

**Supplementary Figure 2.** Total protein was harvested from 3 different clones of the HEK-DHX29^active^ cell lines and used for western blot analysis. Membranes were blotted using antibodies against DHX29 and tubulin.

**Supplementary Figure 3.**

HEK293-NT1 KO cells were incubated for 24 hrs with L/LWRN C.M. Total protein was harvested for Western Blot analysis. Membranes were blotted using antibodies against non-phospho(active) β-catenin, cyclin-D1 and tubulin.

**Supplementary Figure 4.**

The HEK293-Hygro^r^ cell line was transduced with part A of the GeCKO library. The transduced cell line and an untransduced control sample were selected with 150µgr/mL Hygromycin for 10 days. Bright-field pictures were taken on day 10.

**Supplemental Data Set Captions**

Data Set S1. MAGeCK scored and ranked list of genes.

Data Set S2. MAGeCK normalized read counts of sgRNAs.

Data Set S3. MAGeCK count summary.
