# Supplementary material for: A CRISPR knockout screen reveals new regulators of canonical Wnt signaling
Source: Oncogenesis. 2021 Sep 22;10(9):63. doi: 10.1038/s41389-021-00354-7 (PMC8458386; doi:10.1038/s41389-021-00354-7)
Supplement: Supplementary file 2 — supp. figs [file 41389_2021_354_MOESM2_ESM.pptx]

## Slide 1
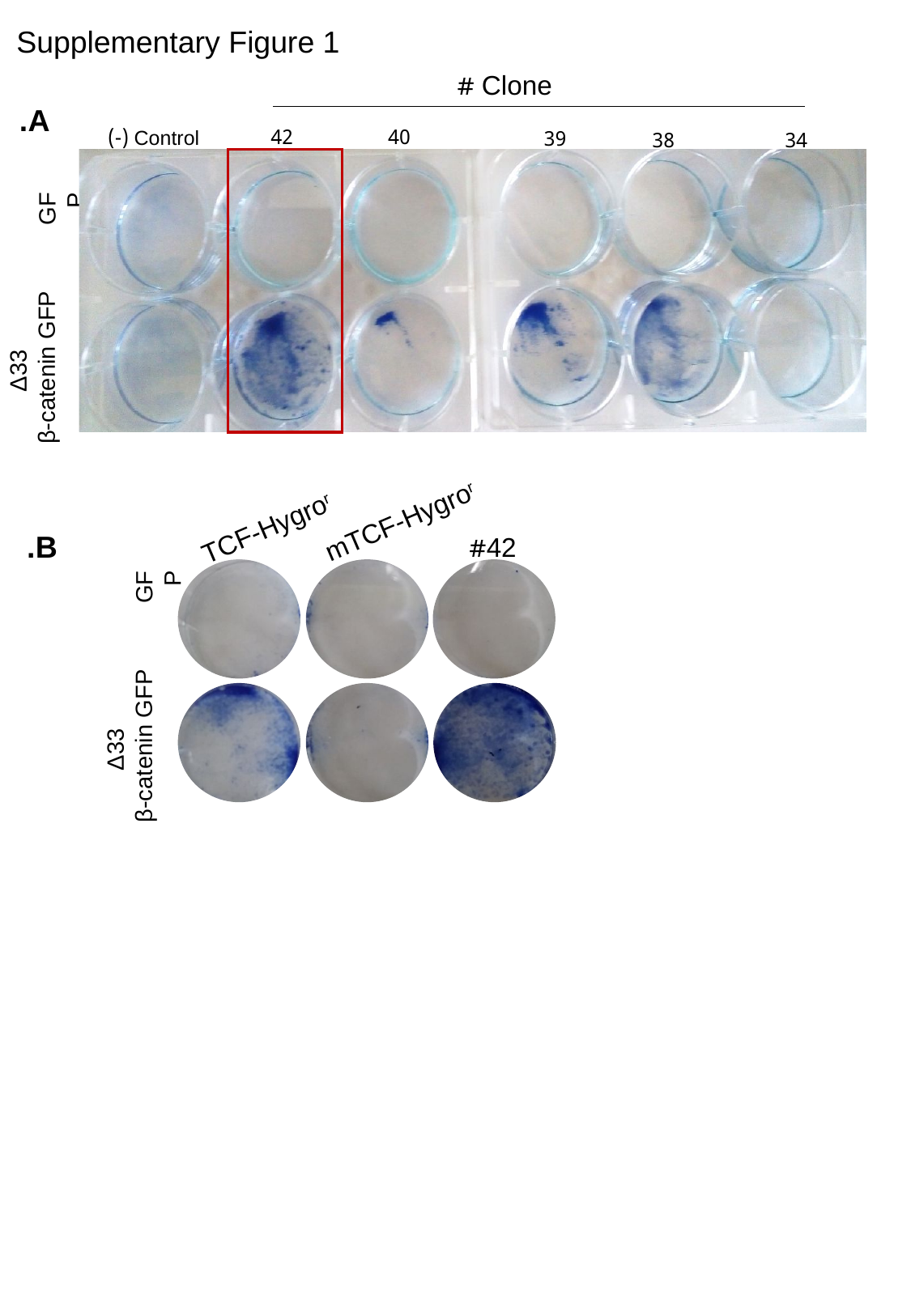

Supplementary Figure 1
Clone #
A.
 40
 42
Control (-)
 39
 38
 34
GFP
Δ33
β-catenin GFP
mTCF-Hygror
TCF-Hygror
B.
#42
GFP
Δ33
β-catenin GFP

## Slide 2
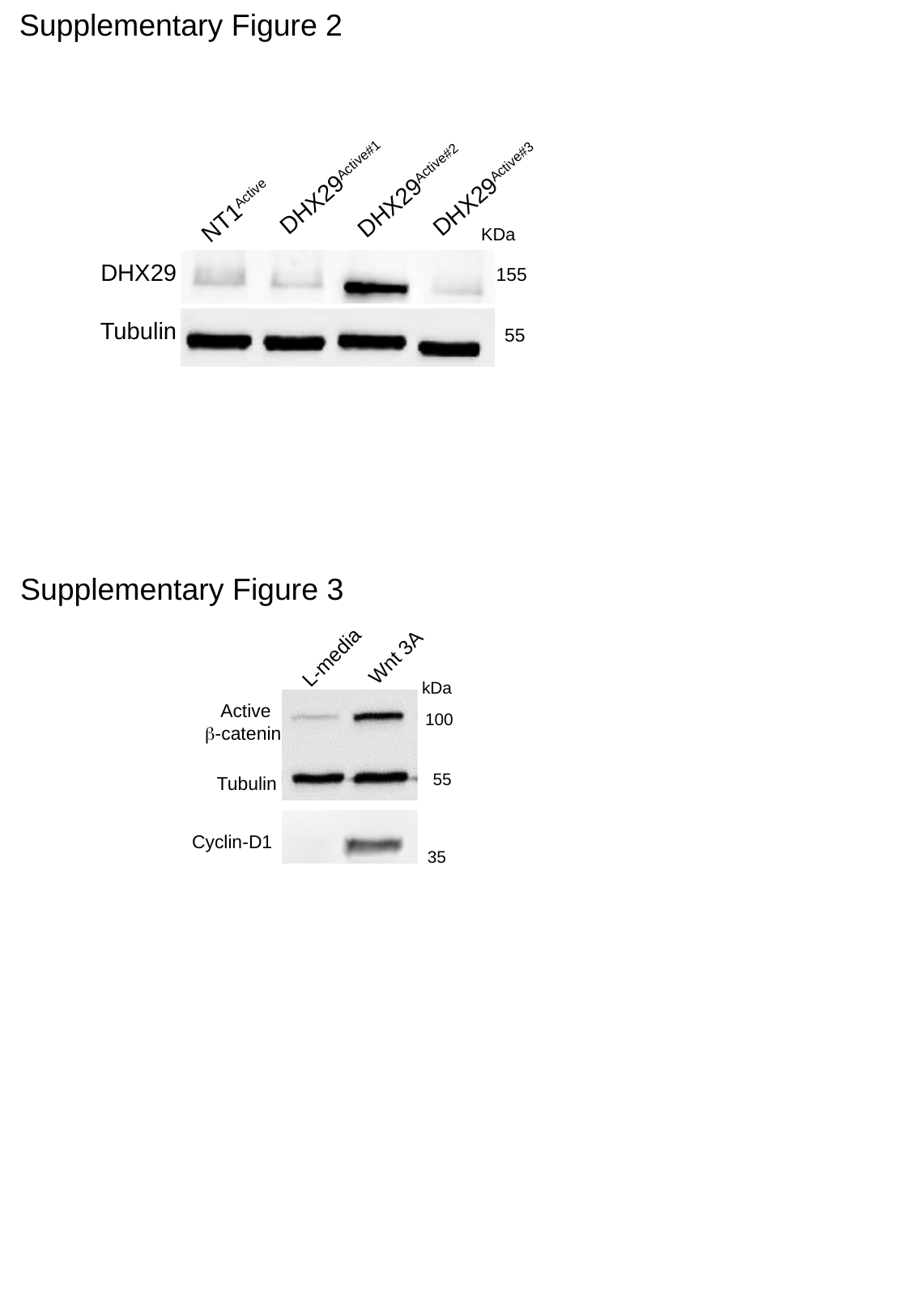

Supplementary Figure 2
DHX29Active#1
DHX29Active#3
DHX29Active#2
 NT1Active
KDa
DHX29
155
Tubulin
55
 Supplementary Figure 3
Wnt 3A
L-media
kDa
Active
b-catenin
100
55
Tubulin
Cyclin-D1
35

## Slide 3
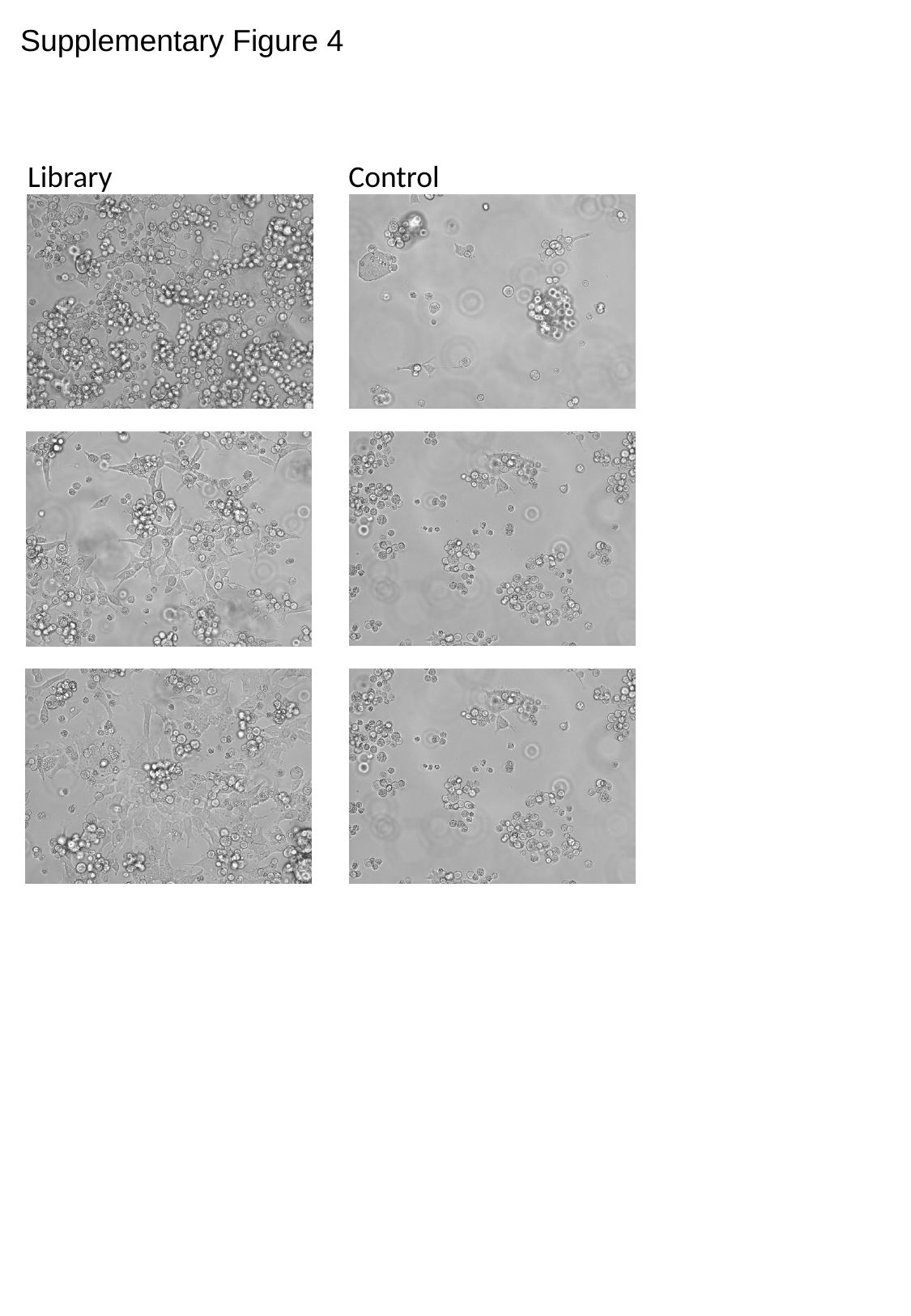

Supplementary Figure 4
Library
Control
